# Mr. Gladstone as an Object Lesson

**Published:** 1888-11-17

**Authors:** 


### The Hospital

A Weekly Institutional Journal of

**Science, Medicine, Nursing, and Philanthropy.**

*For Hospitals, Asylums, Medical Practitioners, Students, Nurses, Families, Parishes, Congregations, and the Charitable Public.*

Vol. V. No. 112.] [November 17, 1888.

### Mr. Gladstone: An Object Lesson.

Long life is not always an unmixed blessing. Some people think it is not a blessing at all. Few old people are happy—many are unhappy; and some are intolerable burdens to their nearest friends. It is hard that old age should not bring content and rest. Mid-life is associated with such stress and strain that one cannot help wishing for every old man and woman a few years of quietness and pleasant peace. Is it a part of the "constitution and course of nature" that advancing age shall have all the disabilities and drawbacks of human life without any of the compensations? If it be, there is a natural instinct in man which tells him that the "constitution and course of nature" is by no means so wise and just as it might be, and would not be any the worse for a good deal of modification. But it is not the natural order of things for old age to have all the bitters and none of the sweets. The cup of life is very mixed no doubt; and many men, who insist upon having nothing but sweets in youth and middle age, find the dregs when they come to the bottom very bitter. But there are other men who keep the cup so well stirred throughout the whole of life that each portion has its share, and no more than its share of sweets and bitters. To such old age is at least as good, and may be better than any other period. For such long life is undoubtedly a blessing to be both desired and sought after.

For effective instruction in any subject nothing is better than a concrete example. Probably more people have taken to thinking of old men during the last few years than has been the case for many generations. There is among us at the present time one of the most striking instances of robust physical energy, high courage, intellectual capacity, and power of endurance in old age which any epoch has known. Mr. Gladstone is a wonder to all his contemporaries—to friends and foes alike. What is the reason, if there be only one reason, or what are the several reasons why he at seventy-seven is able to stand more mental and physical strain and to do more work with less fatigue and injury to health than the majority of men at fifty-seven? Most people will answer at once that his present health is the resultant of his splendid constitution, his well-ordered habits, his physical exercise, and his easy circumstances. That is no doubt largely true. But many a man has had all the natural advantages of Mr. Gladstone, and yet at seventy-sevenhas only been fit for a bath chair, nursery diet, and a private medical attendant. The explanation of Mr. Gladstone's present position is an intellectual one. He has had the brains to see and to understand what he could do and what he could not do. He has comprehended those practical physiological problems that show which way health lies; and he has had the strength of will to carry out in practice, not now and again, but always and constantly, the principles and rules which alone can lead to health. The explanation of Mr. Gladstone's health is, in ultimate analysis, clearness of intellect and force of character.

There are few men who do not know that plain living is better than rich and heavy feeding. Yet what numbers habitually indulge in all kinds of dishes they cannot digest. What thinking man will refuse to admit that strict temperance in alcoholic drinks is conducive to the highest physical wellbeing? But how many will deny themselves systematically that extra quantity of wine, or spirits, or beer, which makes the difference between strong and defective health? It is one of the most elementary commonplaces, not of the physicians consulting-room only, but also of the home and of the club, that a reasonable amount of physical exercise every day is absolutely indispensable to even a moderate degree of health. But does one city man, or clergyman, or lawyer, or even doctor, in a dozen exercise every part of his body a tenth part as much as it should be exercised? Certainly he does not. All these things we all know as well as Mr. Gladstone. The difference is that whereas we know them and do not do them, or do them by fits and starts, he has known them and done them, done them regularly, and done them with all his heart and soul. In this last part of the sentence lies the whole root of the matter: the carrying out of rules of health, not only with regularity and persistence, but also with all the heart and mind The best of rules carried out mechanically and in the spirit of a drudge yield results which are also of the mechanical and drudgery kind. Rules must be inspired with the intensest life and soul, if they are to yield fruits of vitality, energy, and delight.

The value of a life like that of Mr. Gladstone, from a physiologist's point of view, is that it is so perfect a combination of the physical and the intellectual, so striking an example of the reward which follows voluntary and perfect obedience to the laws of health. Multitudes of men have broken down and become prematurely old from hard work, who yet have not done a third part of the work accomplished by Mr. Gladstone. How is this? Because they have not worked intelligently enough. They have been in too great haste for results. Tney have tried to force the pace. They have not waited upon their natural possibilities. Instead of working themselves as a wise rider works his horse, they have been like fools in the saddle, who so long as whip and spur can keep their beasts at full pace, so long the full pace is maintained. The consequence of such riding, as every rider to hounds knows, is that the finest-couraged animal in the world will drop dead beneath his foolish and brutal master.

There is no reason to doubt that Mr. Gladstone was born of a tough Scotch family. He inherited a sound constitution. But hundreds of other men have been born with constitutions quite as good. It seems certain, too, that he never impaired his vitality by youthful vices. That is a circumstance of the utmost importance in considering the question of long life and an active and prosperous old age. He married early, and early settled down to the serious business of his career. He has been signally blessed in his choice of a helpmeet, and Mrs. Gladstone has had no small share in securing the marvellous results we are considering. Mr. Gladstone's life has been one of constant and strenuous work, No life is so conducive to longevity as one so spent, provided, of course, that the work is the servant and not the master of the worker. Many people are slaves to their work. Such are not likely either to live long, or to be very successful in life. The judicious balancing of physical against mental work, with the power of changing the current of thought at will, and the companion power of almost always commanding sleep: these have been some of the mainstays and buttresses of Mr. Gladstone's practically unimpaired health. For, in spite of his seventy-seven years and his unflagging activity in all directions, the wonderful energy and power of endurance he displayed at Birmingham and Wolverhampton last week show that up to the present moment time has made no serious inroads either upon brain or physical energy. For a man of seventy-seven to speak for an hour and three-quarters at a stretch to nineteen thousand people, and to make himself heard by nearly all of them during all that time was a feat probably without parallel in the history of popular oratory.

Mr. Gladstone's woodcutting has been, and now is, as physiologically judicious as it is artistically picturesque. Probably it has had much more to do with the length and strength of his life than either Sir Andrew Clark or any other physician. The first to admit this would be Sir Andrew Clark himself. If there were to arise in this generation a prophet of physic as there have arisen prophets of morals and of religion, the one burden of his cry would be, "Work for the body and the body for work." The body must work, work daily, work sufficiently and work with purpose and for results, if the highest and best of which a man is capable are always to be got out of him. Whatever may be thought of Mr. Gladstone's politics or other views of life and duty, of this we may all be certain, that he has given to this generation an object lesson in healthy living which all men and all women and all children will do well to learn by heart.

